# ADC Values of Cytologically Benign and Cytologically Malignant 18 F-FDG PET-Positive Lymph Nodes of Head and Neck Squamous Cell Carcinoma

**DOI:** 10.3390/cancers14164019

**Published:** 2022-08-20

**Authors:** Petra K. de Koekkoek-Doll, Sander Roberti, Laura Smit, Wouter V. Vogel, Regina Beets-Tan, Michiel W. van den Brekel, Jonas Castelijns

**Affiliations:** 1 Department of Radiology, The Netherlands Cancer Institute, 1066 CX Amsterdam, The Netherlands; 2 Department of Epidemiology and Biostatistics, The Netherlands Cancer Institute, 1066 CX Amsterdam, The Netherlands; 3 Department of Pathology, The Netherlands Cancer Institute, 1066 CX Amsterdam, The Netherlands; 4 Department of Nuclear Medicine, The Netherlands Cancer Institute, 1066 CX Amsterdam, The Netherlands; 5 Department of Radiation Oncology, The Netherlands Cancer Institute, 1066 CX Amsterdam, The Netherlands; 6 Department of Head and Neck Surgery and Oncology, The Netherlands Cancer Institute, 1066 CX Amsterdam, The Netherlands; 7 Department of Maxillofacial Surgery, Amsterdam University Medical Center, University of Amsterdam, 1012 WX Amsterdam, The Netherlands

**Keywords:** DW-MRI, FDG-PET, real-time image fusion, lymph node, head and neck oncology

## Abstract

**Simple Summary:**

In squamous cell carcinoma of the head and neck, 18F-fluordeoxyglucose positron emission tomography (FDG-PET), diffusion-weighted magnetic resonance imaging (DW-MRI) and ultrasound-guided fine needle aspiration are commonly used imaging tools for nodal staging (N-staging). Although FDG-PET has good performance in nodal detection, it is still difficult to distinguish between PET-positive reactive and malignant nodes for the purpose of selecting nodes to be aspirated. DW-MRI can help to detect small lymph node metastases, and an inverse correlation with FDG uptake is expected. We found a mild negative correlation between SUVmax and ADC. Comparing the apparent diffusion coefficient (ADC) values between PET-positive and PET-negative nodes, ADC was significantly higher in PET-negative nodes. Whereas no significantly lower ADC value of cytological malignant nodes could be found overall, in the subgroup of non-HPV-related nodes, the ADC values of cytologically malignant PET-positive nodes were significantly lower than in cytologically benign nodes. This finding might be helpful in selecting nodes for puncture.

**Abstract:**

Nodal staging (N-staging) in head and neck squamous cell carcinoma (HNSCC) is essential for treatment planning and prognosis. 18F-fluordeoxyglucose positron emission tomography (FDG-PET) has high performance for N-staging, although the distinction between cytologically malignant and reactive PET-positive nodes, and consequently, the selection of nodes for ultrasound-guided fine needle aspiration cytology (USgFNAC), is challenging. Diffusion-weighted magnetic resonance imaging (DW-MRI) can help to detect nodal metastases. We aim to investigate the potential of the apparent diffusion coefficient (ADC) as a metric to distinguish between cytologically reactive and malignant PET-positive nodes in order to improve node selection criteria for USgFNAC. PET-CT, real-time image-fused USgFNAC and DW-MRI to calculate ADC were available for 78 patients offered for routine N-staging. For 167 FDG-positive nodes, differences in the ADC between cytologically benign and malignant PET-positive nodes were evaluated, and both were compared to the ADC values of PET-negative reference nodes. Analyses were also performed in subsets of nodes regarding HPV status. A mild negative correlation between SUVmax and ADC was found. No significant differences in ADC values were observed between cytologically malignant and benign PET-positive nodes overall. Within the subset of non-HPV-related nodes, ADC_b0-200-1000_ was significantly lower in cytologically malignant PET-positive nodes when compared to benign PET-positive nodes. ADC_b0-1000_ and ADCb_0-200-1000_ were significantly lower (*p* = 0.018, 0.016, resp.) in PET-negative reference nodes than in PET-positive nodes. ADC was significantly higher in PET-negative reference nodes than in PET-positive nodes. The non-HPV-related subgroup showed significantly (*p* = 0.03) lower ADC values in cytologically malignant than in cytologically benign PET-positive nodes, which should help inform the node selection procedure for puncture.

## 1. Introduction

Head and neck squamous cell carcinomas (HNSCC) account for around 4% of all malignancies [1]. The presence of cervical lymph node metastases reduces the expected survival rate by approximately 50%, especially in HPV-negative tumors [2]. Therefore, nodal staging (N-staging) in HNSCC is essential for the assessment of prognosis and treatment planning [3]. Clinical examination of the neck detects around 60–70% of metastases [4], which means that about 30–40% of lymph node metastases are missed.

Medical imaging plays a major role in the detection of those clinically occult metastases. Computed tomography (CT), magnetic resonance imaging (MRI), 18F-fluordeoxyglucose (FDG) positron emission tomography combined with computed tomography (PET-CT) and ultrasound-guided fine needle aspiration cytology (USgFNAC) are commonly used for this purpose. In clinically node-negative necks (cN0), the pooled estimates for sensitivity were reported as 52% (95% confidence interval (CI), 39–65%), 65% (34–87%), 66% (47–80%) and 66% (54–77%) on a per-neck basis for CT, MRI, PET and USgFNAC, respectively [5]. For N-staging with USgFNAC, node selection plays a major role in obtaining high sensitivity, since specificity for FNAC is 100% [6]. In a recent meta-analysis, USgFNAC was found to be the most accurate imaging modality for detecting cervical lymph node metastases [7]. With morphologic imaging, size is one of the most important criteria for node selection, in addition to necrosis, irregular enhancement and signs of extra nodal spread [8]. Although size is important, reactive lymph nodes might also be enlarged and small lymph nodes can contain micro metastases. To differentiate between reactive nodes and metastatic nodes, a minimal axial diameter between 8 and 12 mm has been established as suitable [9,10]. To minimize the risk of overlooking small metastases, small lymph nodes should also be aspirated, i.e., a low cut-off value should be used. However, this will lead to a higher rate of unnecessary lymph node punctures [11].

Functional imaging techniques such as Doppler sonography, PET-CT and diffusion-weighted imaging (DWI)-MRI as an additional modality can provide information concerning the underlying biology/pathology of the imaged lesion. It has been shown that assessing peripheral vascularization with power Doppler sonography is the best sonographic feature to predict malignancy in cervical lymph nodes [12].

18F-FDG PET-CT has good diagnostic performance, although it overlooks 50% of small metastases in cN0 necks [13], and small PET-positive lymph nodes can have normal ultrasound features; real-time fusion would help to recognize those nodes on ultrasound. In a recent study, we showed that real-time image fusion to guide USgFNAC is feasible in head and neck cancer imaging/diagnosis [14]. It remains a notable challenge, however, to distinguish between small PET-positive reactive nodes and nodes with micro-metastases. Using small cut-off values for the maximal standardized uptake value (SUVmax) in order to select nodes for real-time image-fused-USgFNAC will improve the detection rate of malignant PET-positive nodes, but will lead to a high rate of unnecessary punctures [15].

Diffusion-weighted MRI (DW-MRI) is a method of signal contrast generation based on differences in Brownian motion, and evaluates the molecular function and micro-architecture of the human body. DW-MRI contrast reflects the diffusion of water in tissue, which is reduced in tissue with higher cellularity. By performing DWI using different b values, quantitative analyses by apparent diffusion coefficient (ADC) map are possible. This analysis is usually performed automatically. DW-MRI is widely used in oncological imaging. It has been shown that DW-MRI is a promising non-invasive tool to guide treatment selection in patients with peritoneal metastases of colorectal cancer. It is also a promising imaging tool for the assessment of treatment response and for differentiating between tumor and inflammatory changes [16,17]. It has been shown that for nodal staging, DWI has better performance than turbo spin echo MRI, with higher sensitivity (76% vs. 7%) but slightly lower specificity (94% vs. 99.5%) in detecting sub-centimeter nodal metastases [18]. Previous studies have suggested that a DWI node-negative neck could be considered for a wait and see policy [19]. Due to increased cell density in tumors and metastases, Brownian motion and therefore DWI are more restricted, and ADC might consequently be lower. An inverse correlation between FDG uptake and ADC in malignant lymph nodes has been reported [20].

Infection with high-risk human papillomaviruses (HPV) has been implicated in the pathogenesis of HNSCCs, and HPV-related HNSCCs are known to have a better treatment response and prognosis [21]. A higher FDG uptake in HPV-related malignant lymph nodes of the neck has been reported [22]. It has also been shown that HPV-related primary HNSSC tumors have lower ADC values than non-HPV-related tumors [23].

So far as we know, ADC has not been related to FDG uptake in PET-positive real-time image-fused guided FNAC nodes. Using real-time image fusion, we were able to identify PET-positive nodes for USgFNAC, and we can further compare ADC and FDG uptake in cytologically proven benign and malignant nodes. Locations of nodes on MRI can easily be correlated to the same nodes on PET-CT.

The aim of this study was to evaluate the diagnostic potential performance of DW-MRI in PET-positive nodes. We wanted to investigate if DW-MRI could help to distinguish small reactive lymph nodes with FDG uptake from small metastatic nodes since node selection for FNAC in those nodes is still very challenging. We wanted to investigate the different ADC values in all nodes and in the subgroups of HPV-related and non-HPV-related nodes. We also wanted to compare different ADC values evaluated on different ADC maps. The main aim was to investigate the potential of ADC to distinguish between PET-positive cytologically malignant and benign nodes in order to improve selection for aspiration and pretreatment lymph node staging.

## 2. Materials and Methods

### 2.1. Patients

We retrospectively included 78 patients with either histopathologically proven HNSCC or lymph nodes proven to be SCC metastases of an unknown primary, and with available nodal staging based on real-time PET-CT-image-fused guided FNAC (Table 1).

For all patients, FDG PET-CT and DW-MRI were present. To identify PET-positive nodes, PET-CT was real-time fused with ultrasound (US), and Fused-USgFNAC was performed. To identify the location of the Fused-USgFNAC nodes on MRI, MRI was (visually) correlated with PET-CT and Fused-USgFNAC. Because of the quality of the ADC map on MRI, ADC measurements of these Fused-USgFNAC nodes were only performed in levels I–III.

All retrospective medical data/biospecimen studies at the Netherlands Cancer Institute have been executed pursuant to Dutch legislation and international standards. Prior to 25 May 2019, national legislation on data protection was applied, as well as the International Guideline on Good Clinical Practice. From 25 May 2019, we also adhered to the General Data Protection Regulation (GDPR). Within this framework, patients are informed and have always had the opportunity to object or actively consent to the (continued) use of their personal data and biospecimens in research. None of the patients included in this study objected to the use of their data. This study was approved by the Institutional Review Board (IRBd20-126).

### 2.2. FDG PET-CT Imaging

FDG PET-CT images were acquired using a Gemini TF scanner (Philips, Bel Air, MD, USA). Patients were prepared according to European Association of Nuclear Medicine (EANM) guidelines and had to fast for 6 h prior to FDG administration. For patients with diabetes mellitus, the plasma glucose level was required to be <10 mmol/L. A dose between 190 and 240 MBq [18F]-fluorodeoxyglucose (FDG) was administered depending on body mass index (BMI). PET images of head and neck were acquired at 3 min per bed position with a total field of view (FOV) of 576 mm (three bed positions), and reconstructed to 2 mm isotropic voxels using a BLOB-OS algorithm including time-of-flight information. For anatomical orientation and attenuation correction, low-dose CT was acquired with 40 mAs and a slice thickness of 2 mm. All FDG PET/CT images were assessed by dedicated nuclear medicine radiologists in the clinical setting; these reports were used for this study.

### 2.3. DW-MRI Imaging

Images were acquired on a 3T Achieva dStream scanner, Ingenia 3T or an Achieva Intera 1.5 T (Philips Healthcare, Best, The Netherlands), using a sensitive-encoding head and neck coil. For all patients, conventional MRI of the entire neck was performed.

Axial fat-suppressed T2-weighted turbo spin-echo MRI (TR/TI/TE 8458/180/20 ms), with 3 mm slice thickness, axial T1-weighted spin-echo MRI (TR/TE 799/10) and gadolinium-enhanced T13D (TR/TE 8.8/4.6 ms) with 1 mm slice thickness were performed. DWI was performed before contrast-enhanced T1-weighted MR imaging. Images were obtained in the axial plane with an echo-planar imaging sequence: TR/TE, 4583/76 ms; b-0 (1 averages), b-200 (2 averages) and b-1000 (4 averages) s/mm^2^; field of view, 230 mm; matrix size, 112 × 87 pixel; slice thickness, 4 mm; no interslice gap; number of signals, 8; acquisition time, 3:25 min. Parallel imaging techniques (SENSE) with a reduction factor of 2.5 were used. ADC maps were generated automatically on the operating console from concurrent images. For some patients, MRI was performed with DWI b-0 (1 average), b-100 (1 average), b-300 (1 average), b-500 (2 averages) and b-800 (2 averages) s/mm^2^; TR/TE, 4333/77 ms; field of view, 250 mm; matrix size, 114 × 101 pixel; slice thickness, 3 mm; no interslice gap; and an acquisition time of 1:06 min.

**Figure 1:**
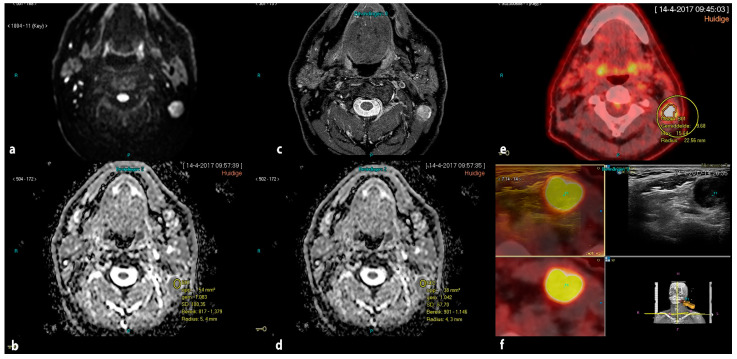
Drawing manual ROI in ADCb_0-1000_ and ADC_b0-200-1000_ in a PET-positive lymph node. (**a**) DWI acquired by b-0-200-1000 s/mm^2^; (**b**) ROI in ADC_b0-1000_; (**c**) axial STIR; (**d**) ROI in ADC_b0-200-1000_; (**e**) corresponding PET-positive node; (**f**) corresponding real-time image-fused guided FNAC of the PET-positive node.

ADC measurements were performed blinded from pathological results by a single radiologist with more than 10 years’ experience in head and neck radiology (PKD) on a PACS workstation (Carestream). To identify PET-positive nodes that underwent Fused-USgFNAC, PET-CT and MRI were visually correlated in an axial and coronal view. Regions of interest (ROIs) were manually drawn and the minimum ADC value was assessed. We placed the ROI in the area of the visually determined lowest signal on the ADC map. In nodes with necrosis, the ROI was placed in the solid part. To avoid partial volume effects from the surrounding tissue, ROIs were placed within the node borders. In the case of small nodes, we placed the ROI in the whole of the node inside the borders. ADC values were obtained not only from ADC_b0-1000_, but also ADCb_0-200-1000_ or ADC_b0-100-300-500-800_, depending on available DW images. (Figure 1).

To determine a reference value for PET-negative nodes, ADC values were also obtained in PET-negative lymph node, with no visible FDG uptake, for each patient.

### 2.4. Ultrasound, Real-Time Image Fusion with FDG PET-CT and Real-Time Fused Guided FNAC

Ultrasound, image fusion with FDG PET-CT and Fused-USgFNAC was performed by using an EpiQ7 G US device (Philips Medical Systems, Bothell, WA, USA), with either an L12-5 or eL18-4 probe.

All US (gFNAC) procedures were performed by a radiologist with 10+ years of US experience in head and neck radiology (P.K.d.K.-D.). The Percunav setup (Philips Medical Systems, Best, The Netherlands) was used according to the manufacturer’s manual. The eL18-4 probe has an integrated tracker; for the L12-5 probe, a bracket and an electromagnetic tracker were added. A patient reference tracker was placed on the forehead, and a field generator was positioned above the patient’s neck. Fusion of the imported FDG PET and CT data took place in the same ultrasound device [14].

After the initial fusion, nodes that were reported as suspicious on FDG PET-CT were marked with the target planning tool and selected for analysis using Fused-USgFNAC. All punctures were performed with a 21G needle. For all nodes that received USgFNAC, the SUVmax values were measured by the radiologist who performed the US (P.K.d.K.-D.), using dyna-CAD and manual drawing of ROIs.

### 2.5. Pathology

Cytological results in nodes for which Fused-USgFNAC was performed were refereed as reference standard. Part of the FNAC material was processed in smears, air-dried and stained with Giemsa stain. Another portion of every aspirate was fixed in 10 mL 4% formalin and embedded in paraffin for further immunohistochemistry examination if deemed necessary, according to routine diagnostic workup. All samples were evaluated by experienced head and neck pathologists in a clinical setting and the cytological results were used retrospectively. HPV status was assessed immunohistochemically on formalin-fixed paraffin-embedded tissue samples from tumor biopsies or resections during standard routine diagnostic procedures. Antibodies for p53 (DO-7, 1/7000, DAKO) and p16 (E6H4; ready to use, Ventana Medical systems/Roche/Arizona, USA) were used in a Benchmark ULTRA autostainer (Ventana Medical systems). Reactions were detected using the OptiView DAB Detection kit (#760-700; Roche) for visualization of p16 and p53. Finally, the slides were counterstained with Hematoxylin II and Bluing Reagent (Ventana Medical Systems).

### 2.6. Statistical Analysis

Analyses were performed using nodes with a sufficient cytological result and clear identification on MRI. We determined the mean ADC of the calculated minimum and mean ADC values for cytologically benign and cytologically malignant nodes. To assess the difference in mean ADC value between cytologically benign and cytologically malignant nodes, accounting for inter-patient correlation, we used a linear mixed effects model with ADC as the dependent variable, malignancy as the independent variable, and a random intercept for patients, fitted with restricted maximum likelihood. Significance testing was conducted using a t-test for the malignancy variable. Differences between mean ADC values for cytologically benign or malignant nodes and PET-negative reference nodes were assessed using a linear mixed effects model with the difference as the dependent variable, no independent variables besides an intercept (fixed effect), and a random intercept for patients, fitted with restricted maximum likelihood. Significance testing was then conducted using a t-test for the (fixed effect) intercept. These analyses were also performed in the subgroups of HPV-related and non-HPV-related nodes. Finally, the overall association between ADC and SUVmax, and between ADC and axial node diameter, was assessed by computing Pearson correlations.

All analyses were performed with R statistical software, version 4.1.1. Missing values were excluded separately for each analysis, all statistical tests were two-sided, and *p*-values below 0.05 were considered statistically significant.

## 3. Results

Real-time image-fused USgFNAC was performed for 140 patients who were referred for N-staging for HNSCC. Patients without PET-positive nodes or without available MRI (*n* = 62) were excluded, leaving 78 patients with a total of 167 PET-positive USgFNAC nodes from levels 1–3 for analysis. The mean age among patients was 62.9 years (range 35–88 years, standard deviation (SD) 10.5). The mean minimal axial diameter for all nodes was 10 mm (range 3–34 mm; SD 6.3). Of the 167 nodes, 91 were cytologically malignant, while the other 76 were cytologically benign. The mean minimal axial diameter was 14 mm (range 3–34 mm; SD 6.9) and 7 mm (range 3–15 mm; SD 2.1) for cytologically malignant and benign nodes, respectively.

Minimum and mean ADC values of DW images were obtained with b-values 0–1000 s/mm^2^ for 155 nodes, and for 154 of these nodes additionally with b-values 0-200-1000 s/mm^2^. For the 12 nodes without b-values 0–1000 s/mm^2^, the minimum ADC was obtained with b-values 0-100-300-500-800 s/mm^2^. The mean value of minimum ADC in cytologically malignant nodes was 0.444 × 10^−3^ mm^2^/s (SD 0.186), 0.645 × 10^−3^ mm^2^/s (SD 0.188) and 0.625 × 10^−3^ mm^2^/s (SD 0.199) for ADC_b0-100-300-500-800_, ADC_b0-1000_ and ADC_b0-200-1000_, respectively. The mean value of mean ADC in cytologically malignant nodes was 0.721 × 10^−3^ mm^2^/s (SD 0.229), 0.834 × 10^−3^ mm^2^/s (SD 0.206) and 0.817 × 10^−3^ mm^2^/s (SD 0.185) for ADC_b0-100-300-500-800_, ADC_b0-1000_ and ADC_b0-200-1000_, respectively (Table 2 and Table 3).

For cytologically benign nodes, the mean minimum ADC values were 0.562 × 10^−3^ mm^2^/s (SD 0.179), 0.625 × 10^−3^ mm^2^/s (SD 0.201) and 0.617 × 10^−3^ mm^2^/s (SD 0.174), for ADC_b0-100-300-500-800_, ADC_b0-1000_ and ADC_b0-200-1000_, respectively. The mean values of mean ADC were 0.842 × 10^−3^ mm^2^/s (SD 0.154), 0.847 × 10^−3^ mm^2^/s (SD 0.201) and 0.806 × 10^−3^ mm^2^/s (SD 0.198), for ADC_b0-100-300-500-800_, ADC_b0-1000_ and ADC_b0-200-1000_, respectively. No significant overall difference in ADC between malignant and benign nodes was observed. In the subgroup of non-HPV-related nodes, there was a significant difference for minimum ADC_b0-200-1000_, with mean values of 0.554 and 0.665 for cytologically malignant and cytologically benign nodes, respectively (*p* = 0.03), and for mean ADC_b0-200-1000_ there was a significant difference, with mean ADC values of 0.780 and 0.923 for cytologically malignant and cytologically benign nodes, respectively (*p* = 0.02). Among HPV-related nodes, no significant differences were observed.

With ADC_b0-1000_, we observed a significantly higher ADC in the PET-negative reference nodes compared to both cytologically malignant PET-positive nodes (minimum ADC difference 0.06, *p* = 0.05; mean ADC difference 0.2, <0.001) and cytologically benign PET-positive nodes (minimum ADC difference 0.10, *p* = 0.02; mean ADC difference 0.10, *p* = 0.004). Only with mean ADC_b0-200-1000_, we observed a significantly higher ADC in the PET-negative reference nodes compared to both cytologically malignant (difference 0.12, *p* = 0.003) and cytologically benign (difference 0.10, *p* = 0.007) PET-positive nodes (Table 4 and Table 5).

**Figure 2:**
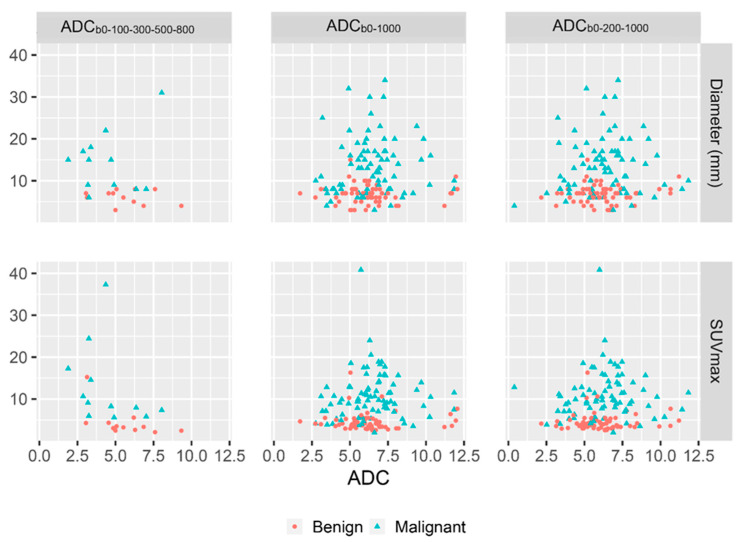
Relationship between ADC and SUVmax, and between ADC and axial node diameter.

Minimum ADC_b0-200-1000_ also differed significantly between the PET-negative reference nodes and benign PET-positive nodes (*p* = 0.02). In the subgroup of HPV-related nodes, minimum ADC_b0-1000_ differed significantly between the PET-negative reference nodes and benign PET-positive nodes (*p* = 0.03), and also mean ADC differed significantly for ADC_b0-1000_ (*p* = 0.01) and ADC_b0-200-1000_ (*p* = 0.01). No strong correlations were observed between minimum ADC and SUVmax or minimum ADC and axial node diameter. A moderate negative correlation was found between SUVmax and a minimum ADC_b0-100-300-500-800_, and mild negative correlation was found for all calculated mean ADC and between all mean ADC and axial node diameter (Figure 2 and Table 6).

## 4. Discussion

Accurate nodal staging is essential for individual treatment planning. With functional imaging, not only anatomical changes, but also metabolic changes in metastases can be detected. As shown in meta-analyses of HNSCC patients, PET-CT has better performance in the detection of metastases than anatomical imaging [24,25]. However, the performance of PET-CT in patients with clinically node-negative neck is poor, with a reported sensitivity of 50–58% [13,26]. Small PET-positive reactive nodes are difficult to distinguish from metastatic nodes, meaning a large number of punctures are requested to improve the sensitivity of imaging.

Due to tumor growth, metastatic nodes often have a higher metabolism and therefore higher FDG uptake. Due to increased cellularity in metastases, DWI is more restricted, which results in a lower ADC.

Significant associations between PET, dynamic contrast-enhanced MRI and DWI parameters have been demonstrated, which indicate a relationship between tumor cellularity, vascular permeability and glucose metabolism in HNSCC [27]. Nakajo et al. demonstrated a significant inverse correlation between FDG uptake and ADC [20]; however, this effect was not observed in other studies [28,29].

In our study, we observed a mild inverse relationship between ADC and SUVmax.

A previous meta-analysis in HNSCC patients showed high diagnostic performance of DW-MRI as a tool to differentiate malignant nodes from benign nodes [30]. Because of real-time image fusion, we were able to compare ADC between truly PET-positive cytologically proven malignant and benign nodes.

Compared to the PET-negative reference nodes, minimum ADC_b0-1000_ and mean ADC_b0-1000_ and mean ADC_b-0-200-100_ were significantly lower for both cytologically malignant and cytologically benign PET-positive nodes.

Although we found a significantly lower ADC in malignant PET-positive nodes compared to PET-negative reference nodes, we did not find a significantly lower ADC in cytologically malignant PET-positive nodes than in cytologically benign PET-positive nodes for the whole group. A possible explanation for the observation that we did not find a lower ADC in cytologically malignant PET-positive lymph nodes when compared to cytologically benign PET-positive lymph nodes could be that in reactive lymph nodes, the primary follicles consisting of loose aggregates of small lymphocytes become secondary or reactive lymphoid follicles with low ADC. These consist of a heterogeneous population of highly proliferative lymphoid cells, follicular dendritic cells and histiocytes that form close cellular interactions [31]. Therefore, a lower ADC might be observed not only in cytologically malignant nodes, but also in cytologically benign PET-positive reactive nodes. Our study suggests that when using DW-MRI, we still have the same problem where we were not able to distinguish small metastatic lymph nodes from reactive (PET-positive) lymph nodes. We found a mild inverse relationship between FDG uptake and ADC values. In a previous study, we were able to show that using a low SUVmax cut-off value can help to improve node selection [15]. It would be interesting to investigate whether we are able to define a cut-off value for ADC values and relationship to SUVmax cut values. A study with a larger number of patients would be required in order to address this point. Another possible explanation could be that some of the cytologically benign cases were (in part) false negative cytologies, with very small metastases.

However, we found significantly lower ADC in non-HPV-related cytologically malignant PET-positive nodes as compared to cytologically benign PET-positive nodes. It has to be mentioned that only a limited number (36) of HPV-related patients were included in our study. To investigate the possible differences between ADC values of malignant and benign nodes in subgroups according to HPV status, a study with a larger number of included patients should be performed.

These significant findings indicate that the measurement of ADC might be helpful to differentiate between small malignant HPV-related PET-positive and reactive PET-positive nodes, and this implies that if a node is PET-positive, then DW-MRI will improve node selection for puncture.

### Limitations

ADC_b0-200-1000_ and ADC_b0-1000_ measurements to calculate ADC values were performed in the same nodes, and for all nodes, results did not differ between ADC_b0-200-1000_ and ADC_b0-1000_, and the use of these two different maps did not affect the outcome. However in the subgroup of non-HPV-related nodes, we found that only in the case of minimum ADC_b0-200-1000_ and mean ADC_b0-200-1000_, a significantly lower ADC in cytologically malignant PET-positive nodes compared to cytologically benign PET-positive nodes could be observed. Since ADC_b0-100-300-500-800_ was only available for 12 patients, we have not been able to show if ADC_b0-100-300-500-800_ would have a better diagnostic performance, but mainly ADC_b0-100-300-500-800_ showed a mild inverse relationship between ADC and SUVmax. It would be interesting to investigate this relationship using a larger cohort of patients.

We did not have histopathological results available in order to have a reference standard, but we did have cytological results of real-time fused USgFNAC. FNAC can produce false negative results. To minimize false negative results, USgFNAC should be repeated and distrusted in nodes predicted to be malignant on PET-FT or MRI. However, it should be borne in mind that very small metastases (micro metastases) cannot really be made visible on either PET-CT or MRI-DWI. Moreover, the cytology in these lymph nodes with small metastases is often either non-diagnostic or (false) negative. Because all FNAC was guided by real-time image-fused PET-CT and US, ADC and SUVmax measurements could take place in well-defined cytologically malignant and benign nodes.

## 5. Conclusions

We found a mild negative correlation between SUVmax and ADC and a significantly higher ADC in PET-negative reference nodes than in PET-positive nodes. In HPV-negative HNSCCs, we found significantly lower ADC values in cytologically malignant PET-positive nodes than in cytologically benign lymph nodes (*p* = 0.03), although this was only observed for one of the ADC modalities and was based on a small number of patients. In HPV-positive tumors, this difference was not significant. In non-HPV-related HNSCCs, DW-MRI might therefore help to select which nodes to aspirate from and might increase the accuracy of FDG PET-CT.

## Figures and Tables

**Table 1:** Diagnosis, number and percentage of patients.

Diagnosis	*n* Patient	% Patient
scc unknown primary	8	10.3%
scc oral cavity	19	24.4%
scc oropharyngeal	32	41.0%
scc hypopharyngeal	4	5.1%
scc laryngeal	8	10.3%
scc nasal cavity paranasal sinuses	3	3.8%
scc nasopharyngeal	2	2.6%
scc cutaneous	2	2.6%
total	78	100.0%

**Table 2:** Difference in minimum ADC between cytologically malignant and benign PET-positive nodes.

ADC		Malignant Nodes		Benign Nodes		Significance
Dataset	Measurement	*N*	Mean (sd)	*N*	Mean (sd)	*p* ^1^
Full	ADC 1 ^2^	12	0.444 (0.186)	12	0.562 (0.179)	0.138
	ADC 2 ^3^	79	0.645 (0.188)	64	0.625 (0.201)	0.620
	ADC 3 ^4^	78	0.625 (0.199)	64	0.609 (0.172)	0.666
No HPV	ADC 1 ^2^	2	0.381 (0.078)	0		
	ADC 2 ^3^	13	0.570 (0.239)	10	0.645 (0.204)	0.086
	ADC 3 ^4^	13	0.554 (0.236)	10	0.665 (0.200)	0.031
HPV	ADC 1 ^2^	2	0.587 (0.163)	4	0.439 (0.157)	0.132
	ADC 2 ^3^	34	0.654 (0.177)	17	0.660 (0.214)	0.996
	ADC 3 ^4^	34	0.640 (0.199)	17	0.605 (0.174)	0.434

^1^ *p*-value based on *F*-test for the factor variable malignancy in a linear mixed effects model with ADC as the dependent variable, and a random intercept for patients. ^2^ ADC1 = ADC_b0-b100-b300-b500-b800_. ^3^ ADC2 = ADC_b0-b1000_. ^4^ ADC3 = ADC_b0-b200-b1000_.

**Table 3:** Difference in mean ADC between cytologically malignant and benign PET-positive nodes.

ADC		Malignant Nodes		Benign Nodes		Significance
Dataset	Measurement	N	Mean (sd)	N	Mean (sd)	*p* ^1^
Full	ADC 1 ^2^	12	0.721 (0.229)	12	0.842 (0.154)	0.400
	ADC 2 ^3^	79	0.834 (0.206)	64	0.847 (0.201)	0.605
	ADC 3 ^4^	78	0.817 (0.185)	64	0.806 (0.198)	0.747
No HPV	ADC 1 ^2^	2	0.602 (0.069)	0		
	ADC 2 ^3^	13	0.780 (0.293)	10	0.920 (0.213)	0.018
	ADC 3 ^4^	13	0.772 (0.298)	10	0.852 (0.199)	0.132
HPV	ADC 1 ^2^	2	0.753 (0.227)	4	0.761 (0.125)	0.256
	ADC 2 ^3^	34	0.842 (0.215)	17	0.819 (0.217)	0.683
	ADC 3 ^4^	34	0.838 0.169	17	0.770 (0.182)	0.174

^1^ *p*-value based on *F*-test for the factor variable malignancy in a linear mixed effects model with ADC as the dependent variable, and a random intercept for patients. ^2^ ADC1 = ADC_b0-b100-b300-b500-b800_. ^3^ ADC2 = ADC_b0-b1000_. ^4^ ADC3 = ADC_b0-b200-b1000_.

**Table 4:** Difference in minimum ADC values between the PET-negative reference node and PET-positive nodes.

ADC		Malignant Nodes			Benign Nodes		
Dataset	Measurement	*N*	Difference ^1^, Mean (sd)	*p* ^2^	*N*	Difference, Mean (sd)	*p* ^2^
Full	ADC 1 ^3^	12	−0.164 (0.291)	0.192	12	−0.089 (0.286)	0.301
	ADC 2 ^4^	79	−0.063 (0.279)	0.050	64	−0.095 (0.275)	0.018
	ADC 3 ^5^	78	−0.044(0.278)	0.083	64	−0.088 (0.277)	0.016
No HPV	ADC 1 ^3^	2	−0.706 (0.078)	0.140	0		
	ADC 2 ^4^	13	−0.236 (0.384)	0.155	10	−0.127 (0.198)	0.275
	ADC 3 ^5^	13	−0.113 (0.273)	0.301	10	0.035 (0.194)	0.601
HPV	ADC 1 ^3^	2	−0.128 (0.152)	0.355	4	−0.169 (0.278)	0.227
	ADC 2 ^4^	34	−0.002 (0.233)	0.721	17	−0.161 (0.377)	0.184
	ADC 3 ^5^	34	0.002 (0.275)	0.637	17	−0.208 (0.280)	0.030

^1^ ADC of node minus ADC of reference node. ^2^ *p*-value based on *F*-test for the factor variable malignancy in a linear mixed effects model with ADC as the dependent variable, and a random intercept for patients. ^3^ ADC1 = ADC_b0-b100-b300-b500-b800_. ^4^ ADC2 = ADC_b0-b1000_. ^5^ ADC3 = ADC_b0-b200-b1000_.

**Table 5:** Difference in mean ADC values between the PET-negative reference node and PET-positive nodes.

ADC		Malignant Nodes			Benign Nodes		
Dataset	Measurement	*N*	Difference ^1^, Mean (sd)	*p* ^2^	*N*	Difference ^1^, Mean (sd)	*p* ^2^
Full	ADC 1 ^3^	12	−0.166 (0.366)	0.201	12	−0.102 (0.196)	0.189
	ADC 2 ^4^	79	−0.151 (0.289)	<0.001	64	−0.102 (0.263)	0.004
	ADC 3 ^5^	78	−0.117 (0.277)	0.003	64	−0.101 (0.288)	0.007
No HPV	ADC 1 ^3^	2	−0.860 (0.069)	0.101	0		
	ADC 2 ^4^	13	−0.258 (0.382)	0.125	10	−0.022 (0.217)	0.953
	ADC 3 ^5^	13	−0.163 (0.347)	0.247	10	−0.013 (0.191)	0.893
HPV	ADC 1 ^3^	2	−0.134 (0.153)	0.341	4	−0.090 (0.067)	0.135
	ADC 2 ^4^	34	−0.099 (0.284)	0.087	17	−0.248 (0.326)	0.010
	ADC 3 ^5^	34	−0.049 (0.252)	0.257	17	−0.255 (0.316)	0.011

^1^ ADC of node minus ADC of reference node. ^2^ *p*-value based on *F*-test for the factor variable malignancy in a linear mixed effects model with ADC as the dependent variable, and a random intercept for patients. ^3^ ADC1 = ADC_b0-b100-b300-b500-b800_. ^4^ ADC2 = ADC_b0-b1000_. ^5^ ADC3 = ADC_b0-b200-b1000_.

**Table 6:** Correlations between ADC and SUVmax, and between ADC and axial node diameter.

Variable 1	Variable 2	Pearson Correlation	Pearson Correlation
		Minimum ADC	Mean ADC
ADC 1 ^1^	SUVmax	−0.45	−0.30
ADC 2 ^2^	SUVmax	0.07	−0.06
ADC 3 ^3^	SUVmax	0.06	−0.04
ADC 1	Diameter	−0.12	−0.18
ADC 2	Diameter	0.07	−0.04
ADC 3	Diameter	0.09	−0.01

^1^ ADC1 = ADC_b0-b100-b300-b500-b800_. ^2^ ADC2 = ADC_b0-b1000_. ^3^ ADC3 = ADC_b0-b200-b1000_.

## Data Availability

The data used to support the findings of this study are available from the corresponding author upon request.
